# Unprecedented tibial bone lengthening of 33.5 cm by distraction osteogenesis for the reconstruction of a subtotal tibial bone defect. A case report and literature review

**DOI:** 10.1186/s12891-021-03950-1

**Published:** 2021-01-18

**Authors:** Abdulnassir Ali, Ying Ren, Chun-Hao Zhou, Jia Fang, Cheng-He Qin

**Affiliations:** 1 Division of Orthopaedics and traumatology, Department of Orthopaedics, Nanfang Hospital, Southern Medical University, Guangzhou, 510515 People’s Republic of China; 2 Department of Nursing, Nanfang Hospital, Southern Medical University, Guangzhou, 510515 People’s Republic of China; 3 Department of Trauma & Orthopaedic Surgery, Guangdong second provincial general hospital, Guangzhou, 510317 People’s Republic of China

**Keywords:** Bone defect, Osteomyelitis, Distraction osteogenesis, Ilizarov technique, Case report

## Abstract

**Background:**

We present a case of an immense unprecedented tibial bone lengthening of 33.5 cm. The management of chronic osteomyelitis of the right tibia with subtotal tibial bone defect, talus defect and equinus ankle deformity. We demonstrate limb reconstruction by distraction osteogenesis and correction of ankle deformity with the Ilizarov technique. Limb salvage was preferred as an alternative to amputation to restore basic limb function.

**Case presentation:**

A 16-year-old male patient fell and injured his right lower leg. He attempted to treat the symptoms with traditional home remedies. During 15 months of self-treating, he developed osteomyelitis of the right tibia and had lost function in his foot. Radiology revealed immense bone defect of the right tibia, including talus bone defect and equinus deformity of the calcaneus. The patient’s right tibia was non weight-bearing, had drainage sinus just below his knee and a large scar anteriorly along the entire length of the tibia.

**Conclusion:**

Upon completion of treatment, the patient was able to avoid amputation of his leg with partially restored function for weight-bearing. He carried himself without assistance after 3 years of lost function in his right leg. Tibial bone distraction osteogenesis of 33.5 cm was done after 90% of the tibial length was defected. To the best of our best knowledge, this case is one of a kind to achieve distraction of tibial bone to such length.

## Background

Chronic osteomyelitis and immense bone defect management still remain a challenge. There are multiple techniques proposed, including distraction osteogenesis, free vascularized fibular graft, and masquelet technique for management of bone defect; however, there is no definitive method or guideline for defect of such magnitude. The patient’s strong emphasis on keeping his leg and avoiding amputation challenges us to meet the requirement. This case is to demonstrate the potential distraction osteogenesis in the management of subtotal bone defect and achieve satisfactory results.

## Case presentation

A 16-year-old boy, after suffering from a falling injury to his right distal tibia, applied home remedies and herbs in an attempt to manage symptoms of swelling and redness. Unfortunately, his skin did not respond well and resulted in the formation of large blisters. He was taken to the local hospital and intravenous antibiotics were given. However, medication was ineffective, and skin around the medial side of right tibia ruptured leaving his bone exposed. He was experiencing repeated rupturing of his wound with purulent discharge that had a foul smell. This later developed to continuous purulent discharge. He was then taken to the local hospital and bacterial culture was done. The results showed *Staphylococcus aureus* and he was eventually transferred to our hospital.

**Fig. 1:**
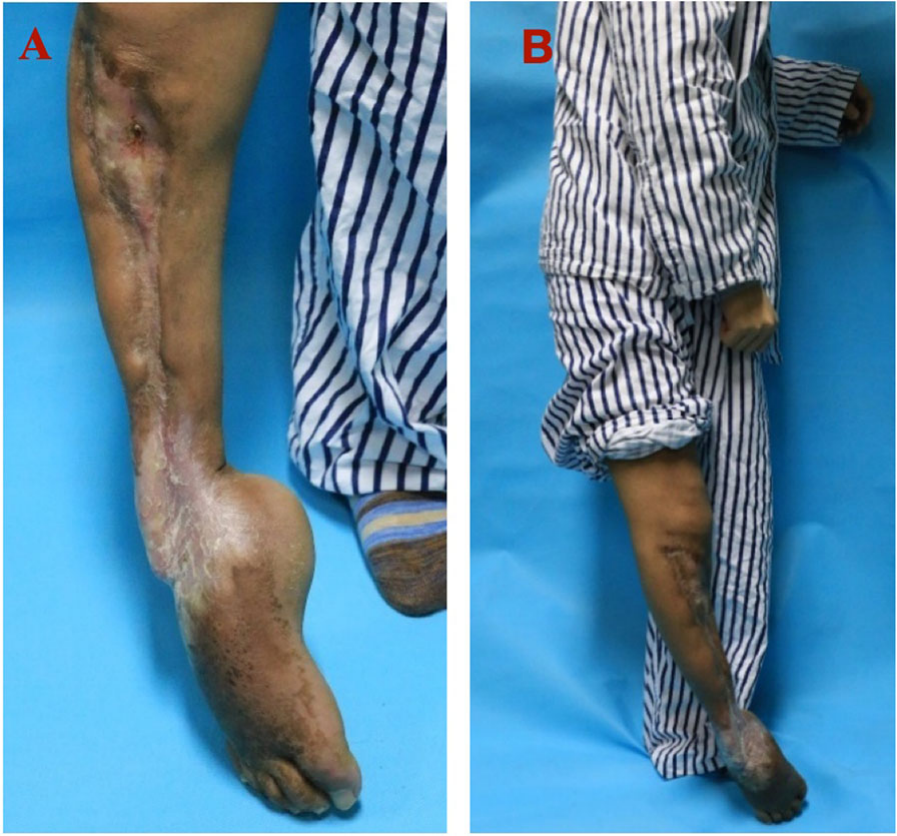
Photo of patient’s infected leg at presentation

**Fig. 2:**
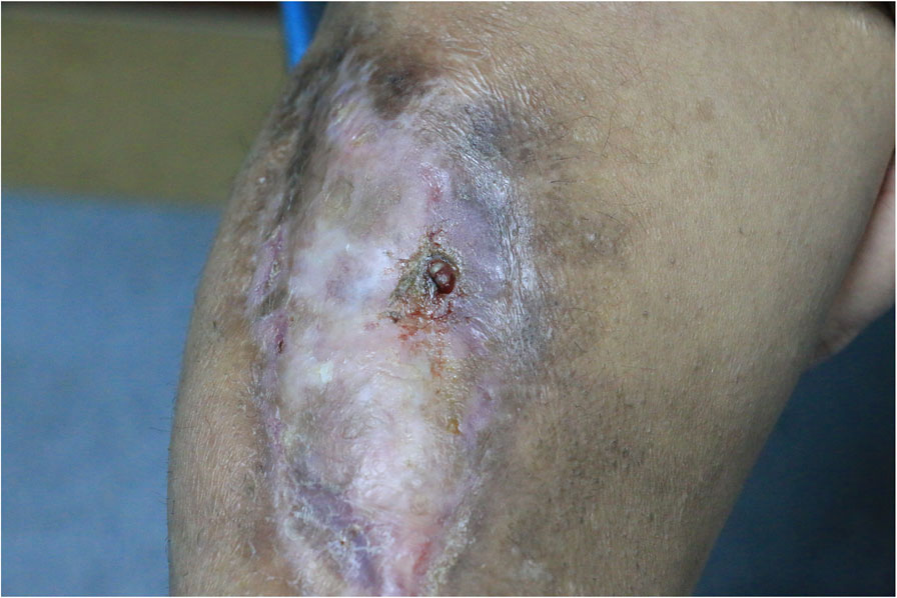
Drainage sinus in the proximal tibia

At presentation, his right foot was not weight-bearing. There was scar tissue along the full length of the anteromedial side of the tibia and fixed equinus deformity and stiffness of the right ankle joint (Fig. 1). There was a draining sinus anteriorly just 4 cm below the knee joint (Fig. 2). On physical examination the right knee flexion was limited to less than 70 degrees, muscles were atrophied, and there was equinus ankle deformity and ankle stiffness. Neurovascular and muscular status of the anterior compartment of the right lower leg was compromised, anterior tibialis was unidentified, dorsal pedis artery was weak compared to the opposite side, right foot lacked sensation on palpation and active dorsiflexion of the foot was completely lost.

**Fig. 3:**
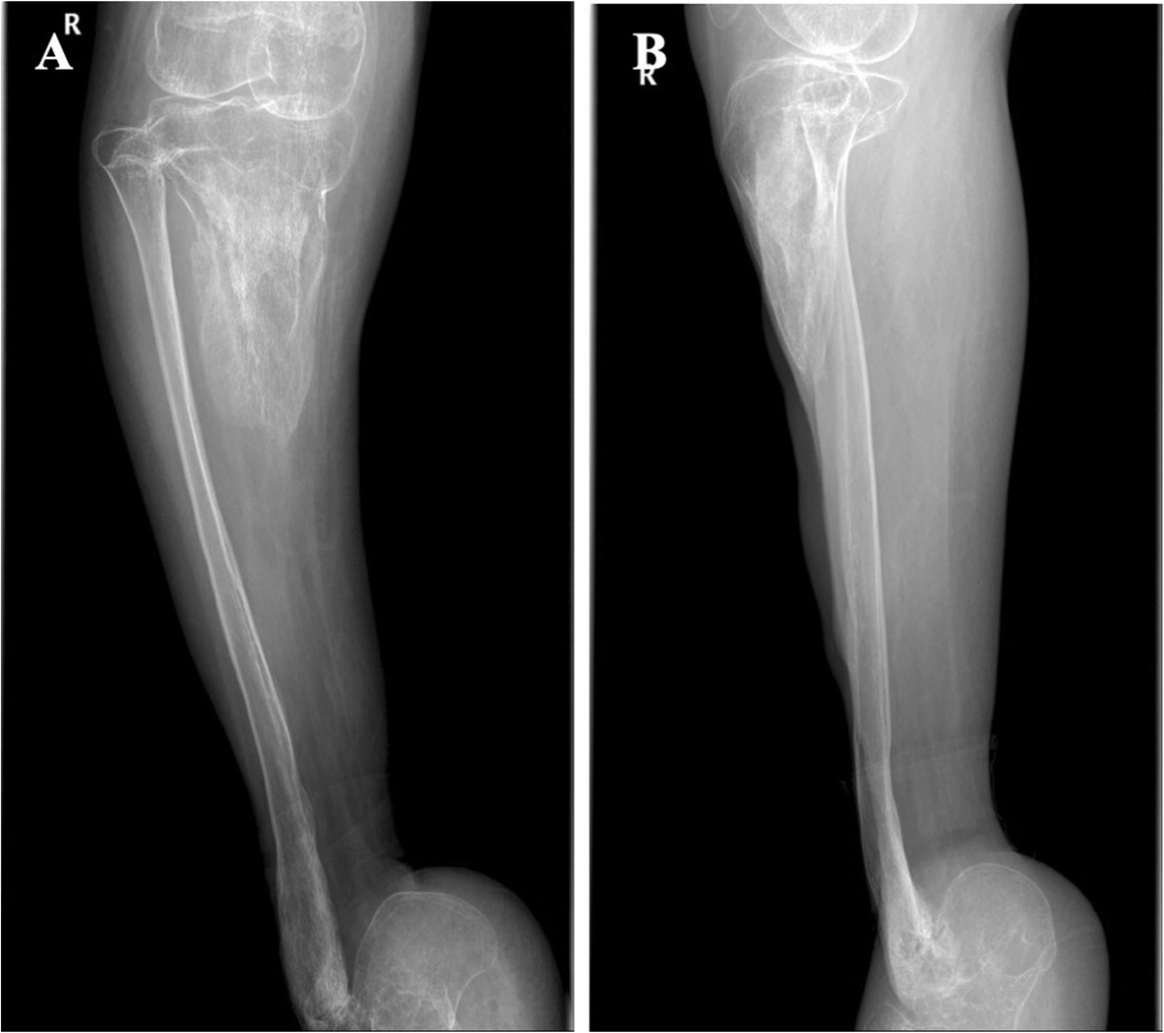
**a**, X-ray AP view of right lower leg. **b**, X-ray Lateral view of right lower leg

**Fig. 4:**
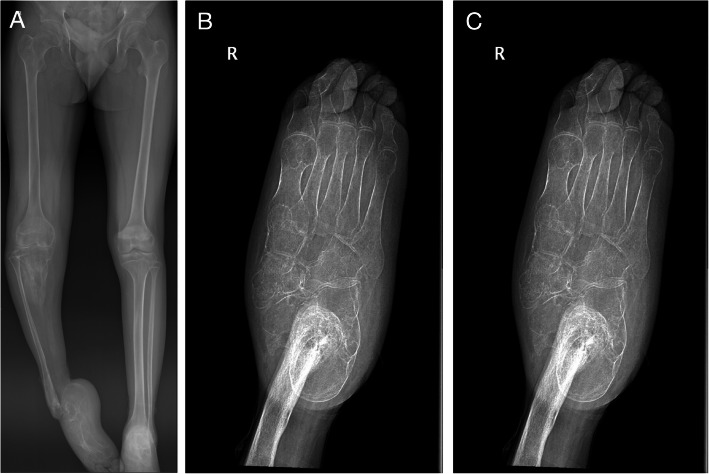
**a**, X-ray AP view bilateral lower limbs. **b**, **c**, X-ray of foot

Radiology showed a tibial bone defect of about 80% of the distal length. The remaining proximal tibia showed some loss of cortex and lytic lesion (Fig. 3). Fibula showed diffused osteomyelitis in the distal part. Furthermore, the X-ray showed talus bone defect and equinus deformity of the calcaneus (Fig. 4).

Laboratory results from blood work revealed ESR 26(mm/1 h), c-reactive protein 6.83(mg/L) and a slightly increased liver function AST/ALT 1.9(IU/L), otherwise normal. A biopsy was taken from the drainage sinus and culture showed the presence of *Staphylococcus epidermidis* and *Pseudomonas aeruginosa*. It was clear that the patient suffered from a severe diffused chronic osteomyelitis of the right tibia according to Cierny-Mader classification type IV class A [1]. The patient and his parents strongly insisted to focus the treatment on preserving his limb.

**Fig. 5:**
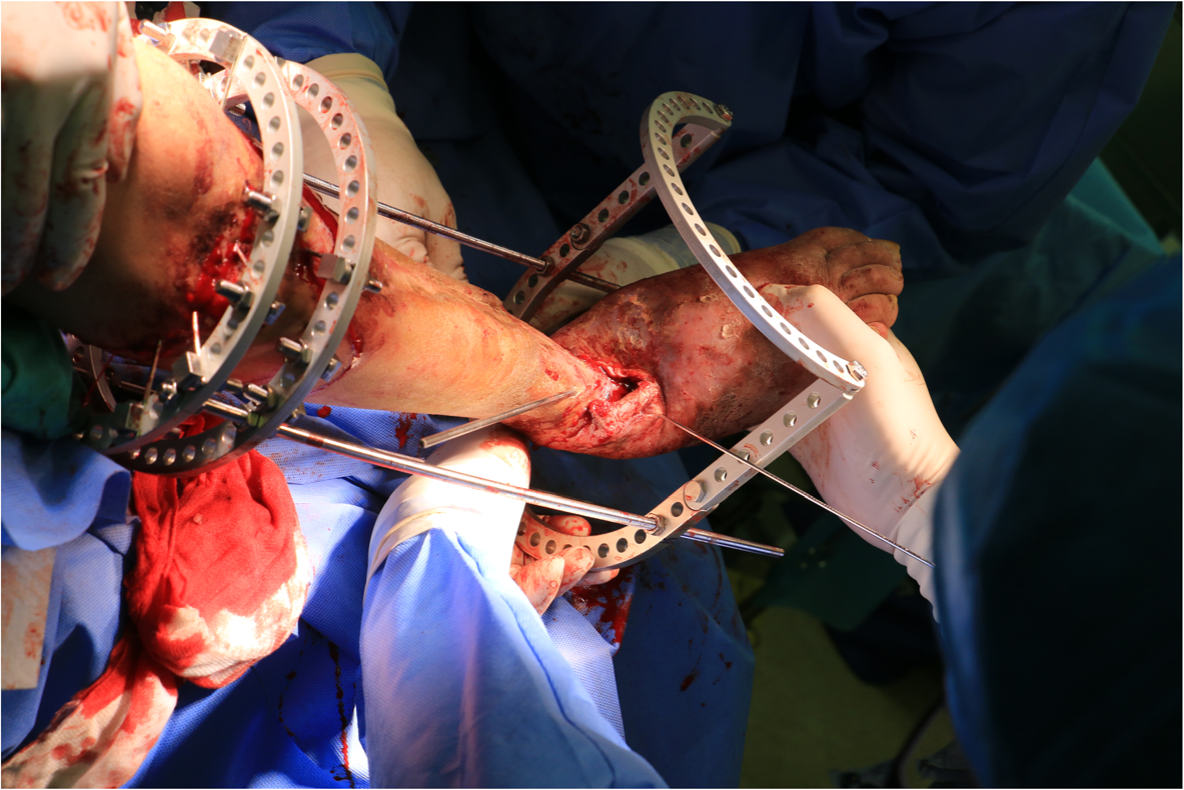
Intra-op photo, alignment of footplate to proximal circular frame

**Fig. 6:**
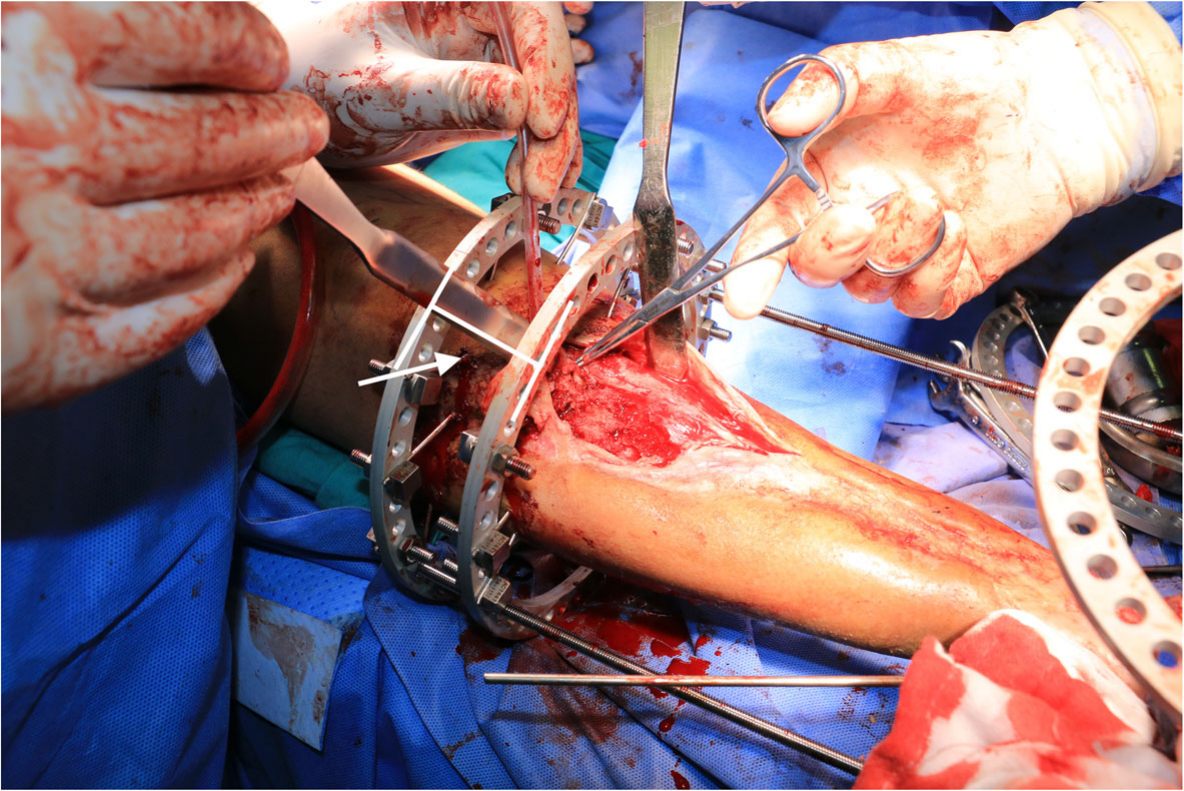
Debridement after circular frame fixation of proximal remains of the tibia. Arrow; length of tibia

**Fig. 7:**
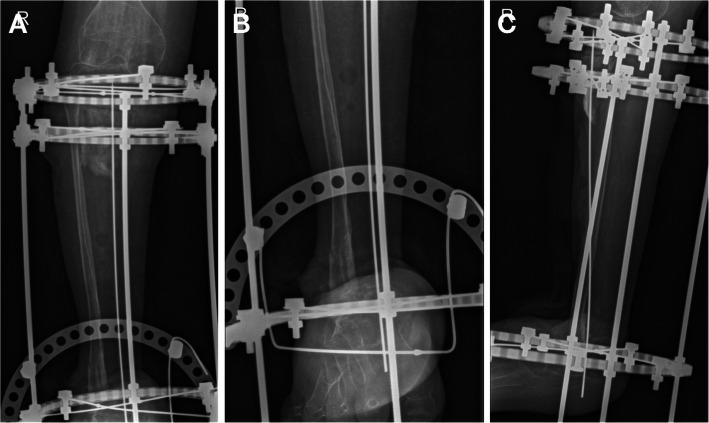
Post-op images of right lower leg. **a**, post-op AP view. **b**, post-op AP view at foot level. **c**, post-op later view

After thorough analysis and planning, the patient was scheduled for his first stage of surgery. We started by excision of the infected part of the distal fibula, followed by application of Ilizarov external fixator for tibiocalcaneal fusion and correction of foot deformity. A footplate was placed on the hindfoot to maintain a neutral foot position (Fig. 5). A long incision was made from the proximal tibia to distal third, three samples for biopsy from deep tissue both bone and scar tissue was collected, and debridement was done while attempting to preserve viable bone from the remains of the tibia (Fig. 6). Followed by repeated irrigation and vancomycin combined calcium sulphate was filled into the cavity then wound was closed (Fig. 7). Intravenous antibiotics were administered for 2 weeks.

A percutaneous tibial osteotomy was not done during the first stage because the remaining proximal tibia was short in length and its proximity to the infected area posed a potential risk of transfer of infection. After the infection n was subsided, the patient was scheduled for second-stage surgery, for percutaneous tibial osteotomy. One week after the procedure, distraction was started at a rate of 0.25 mm, three times per day. This rate was manipulated following callus formation and consolidation. Partial weight-bearing with the assistance of crutches and physiotherapy was advocated for his right knee to prevent knee stiffness. At discharge, the patient was advised to continue his daily distraction and was scheduled for a monthly follow up at the clinic.

**Fig. 8:**
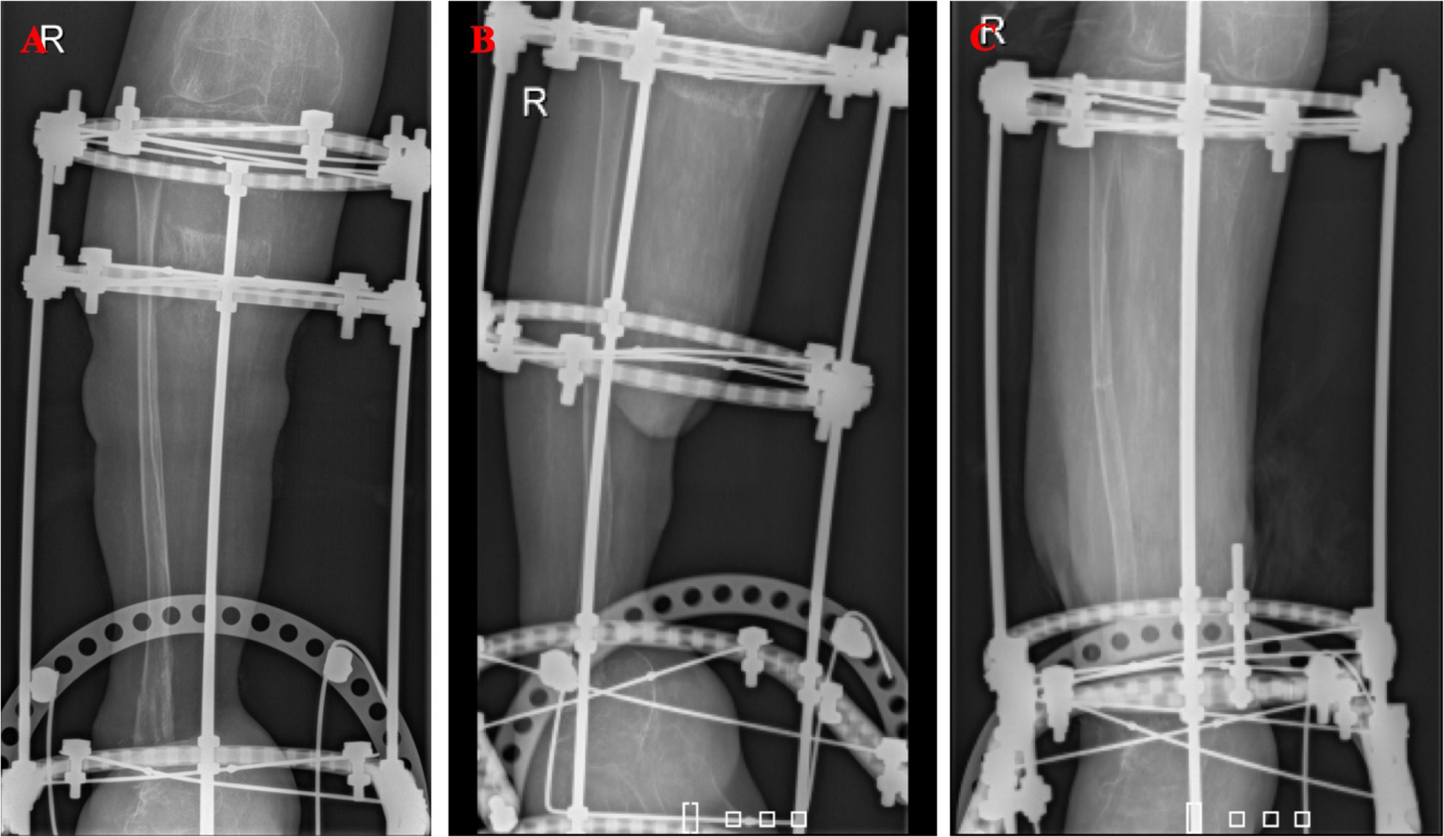
Radiology at 3 months (left), 6 months (middle) and 9 months (right)

**Fig. 9:**
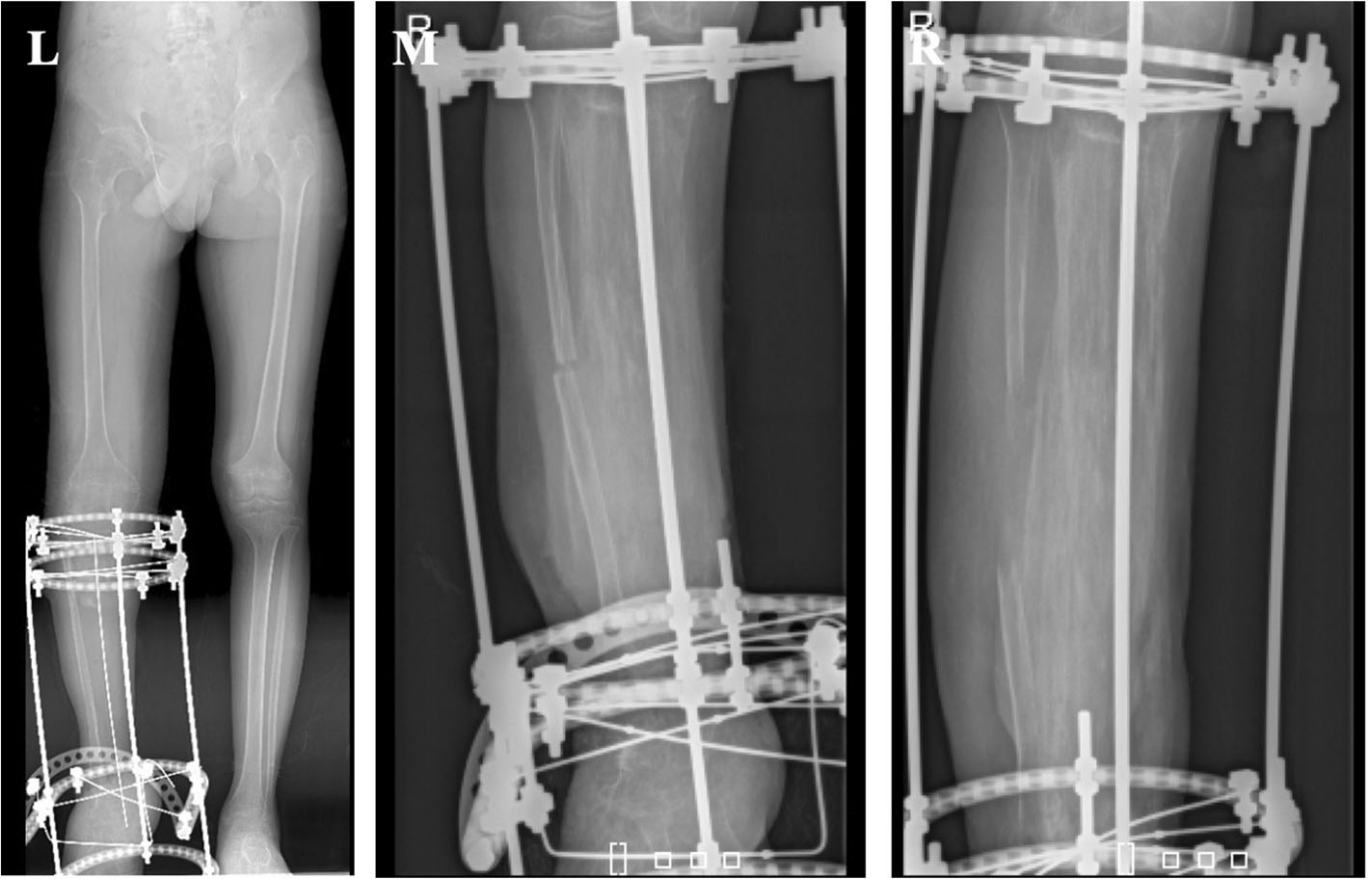
Left, X-ray of bilateral lower limbs showing limb discrepancy. Middle, 10 days post-op fibular osteotomy. Right, 45 days post-op fibular osteotomy

Following the daily distraction and monthly checkups, 9 months after surgery the tibia had grown to fuse with the calcaneus (Fig. 8). The patient was taken for a third surgery for fibular osteotomy to further continue distraction osteogenesis to correct limb discrepancy. During surgery manual compression was done to avoid nonunion in the osteotomy site of the fibula (Fig. 9).

**Fig. 10:**
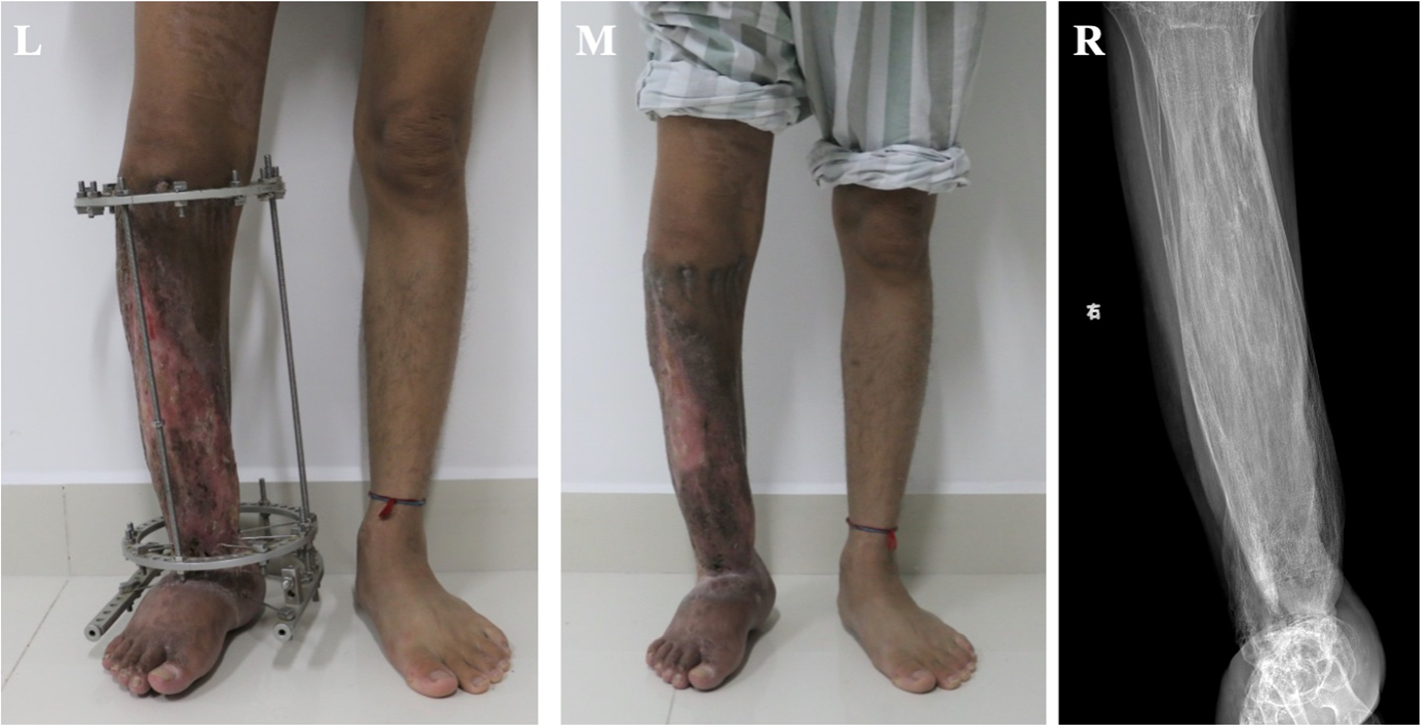
L, photo of bilateral lower legs at completion of distraction. M, photo of bilateral lower legs after removal of apparatus. R, X-ray of newly formed right tibia after removal of apparatus

The skin and scar tissue stretched smoothly with bone distraction. During the 14 months of distraction, the patient showed no further complications and the tibia was lengthened until discrepancy was corrected. The patient was able to walk without crutches, was fully weight-bearing and was advised to keep wearing the apparatus for 3 months to ensure continuous compression until healing was achieved at the docking site (Fig. 10).

Distraction was stopped after reaching the presumed length. Five months later, when sufficient consolidation and healing of the docking site was appreciated on the X-ray and the Ilizarov apparatus was removed. At removal, bone lengthening was recorded at 32 cm. Though the patient lost function in the foot due to the absence of ankle and subtalar joint the patient restored normal gait, he was able to walk without crutches and was able to return to school.

**Fig. 11:**
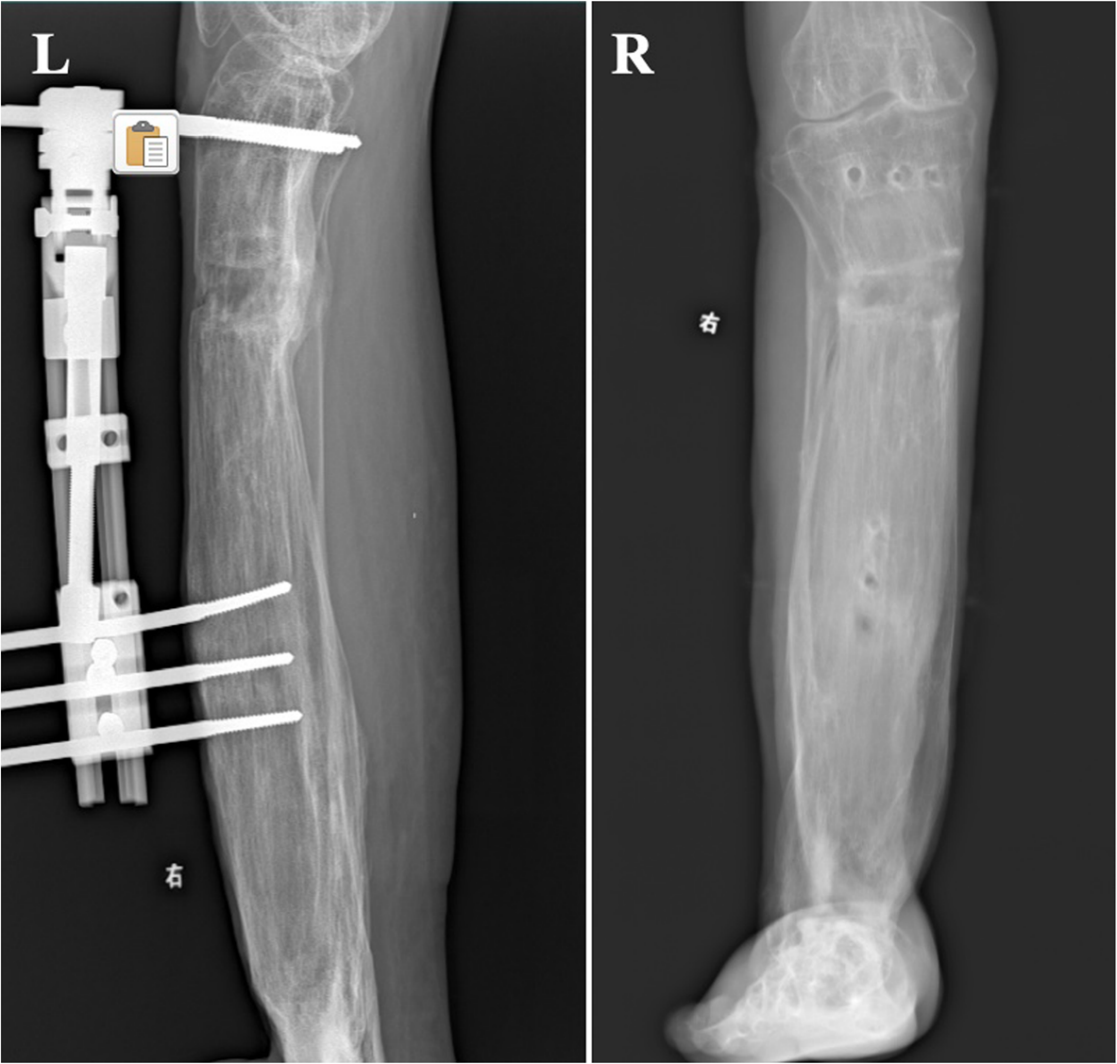
L, lateral view X-ray of right lower leg after completion of valgus correction and distraction. R, AP view X-ray of right lower leg after removal of apparatus

Shortly after, the patient showed slight discomfort with minute discrepancy and varus deformity of the tibia. He was admitted for varus deformity of 15 degrees and discrepancy correction. By using a unilateral external fixator, we corrected the angulation and further distracted the tibia for 1.5 cm, totaling distraction osteogenesis of 33.5 cm (Fig. 11). After the restoration of the full length of the lower extremity and plantar grade foot, the patient was able to walk and even jog without crutches. Functional outcome was evaluated during follow up using Lower Extremity Functional Scale (LEFS) and the patient had a score of 67/80 or 83.8% at his final checkup [2].

## Discussion

There are no reports or guidance on how to deal with such subtotal tibial bone defect and talus defect without considering amputation as the main option of treatment. Numerous techniques have been developed to manage cases involving massive bone defects with various degrees of success. Some showed limited success and others had positive results with a high probability of complications [3–5]. Management of large bone defect still remains a challenge to meet smooth and satisfactory results for both patient and physicians.

Among the various types of techniques in dealing with immense bone defects, it is arguable whether distraction osteogenesis, free vascularized fibular graft, or masquelet technique is a better treatment, or even amputation, with concern to functional result [6–8]. The patient’s health and socioeconomic status also are influential factors to be taken into consideration [9]. Patient compliance itself has a major implication on progress and result [10].

Vascularized fibular graft or Ipsilateral pedicled fibular transfer was not an option; the immense size of the defect and the active infection would significantly increase the risk of avascular necrosis. The fibular graft length and thickness would not satisfy the requirement, increasing the risk of a stress fracture. Hypertrophy of the fibula will take years after fibular transport is complete. The limb discrepancy at presentation in addition to the massive defect appeared inapplicable as the available length of fibula for transfer is 20-24 cm. Furthermore, this procedure would extend the treatment period resulting in a significant increase in hospital cost [9, 11–13]. Bone graft was impractical; even from multiple donor sites, the harvested bone would not be enough to fill the massive void and is associated with morbidity of donor site [6]. Keeping in mind the limb discrepancy next to the tibial defect and talus defect, these options would not be applicable in this case of such immense defect.

Distraction osteogenesis using the Ilizarov technique in many studies has proven to be advantageous over other management options in dealing with massive segmental bone defects whether it be a traumatic bone defect or infected bone defect [3–6, 8, 14]. It allows maneuvering for tackling associated problems like soft tissue defect and complication related to infection [3, 6, 7]. A wide range of angulation management all done externally with the manipulation of the rods sparing patients from invasive intervention [15]. Unfortunately, polyfocal Ilizarov bone transport was not feasible in this case as the remaining proximal tibia was too short. To the best of our knowledge, there is only a single report of bone lengthening with a record of maximum 31.5 cm [16].

Taking into consideration the patient’s age and cultural influence, amputation was not an option considered for discussion. The patient and his family greatly insisted on treatments that would allow him to keep his leg. Most importantly the neuromuscular and vascular status for most parts of his leg encouraged us to take on the challenge, to figure out a way to control the infection, restore a certain amount of function, correct discrepancy, and keep his leg.

We have also incorporated the use of vancomycin loaded calcium sulphate as a means of direct administration of antibiotics, as filler to the dead space and for calcium sulphate’s osteoconductive characteristics. Calcium sulphate has proven to be the standard choice for antibiotic carrier due to its bio-absorbable character [17]. Calcium sulphate also greatly decreases infection recurrence and rarely causes docking site obstruction which otherwise requires additional surgery [18]. Masquelet technique was not suitable for this case. It is a two-stage procedure and it would still need bone grafting. The defect size is extremely huge and would require multiple attempts [19]. The application of calcium sulphate provides further use as an osteogenic component, whilst acting as a dead space filler and an antibiotic carrier [20].

In this particular case, we chose distraction osteogenesis with the use of the Ilizarov technique. The Ilizarov apparatus allowed us to perform multiple procedures namely, excision of the infected portion of the distal fibula, correction of plantar flexion of the foot with the use of footplate, debridement of infected tissue and dead bone, and implantation of calcium sulphate in the first stage surgery. It permitted us to perform a single procedure of percutaneous osteotomy of the proximal tibia for distraction osteogenesis. It also permitted us to perform fibular osteotomy for the third stage tibial lengthening without having to adjust the apparatus. Thus, it reduces the multiple stages of surgery to just 3 stages. The varus deformity may be due to relatively early frame removal and leaving the patient to walk immediately without a period of splinting. The length of distraction osteogenesis is influenced by many factors, mainly by the condition of surrounding soft tissue. There are reports of bone lengthening ranging from 14 cm to 31.5 cm [7, 16, 21, 22]. This case demonstrated a newly grown bone of 33.5 cm in length by unifocal distraction osteogenesis, which to our best knowledge is the first of its kind.

The patient was able to perform full weight-bearing activities without assistance during the second half of the treatment period and after the removal of the apparatus. Relatively normal gait was restored and the patient was able to return to school, making a significant impact on his living standard.

## Conclusion

A full lengthening restoration of the tibia by unifocal lengthening amounting an unprecedented 33.5 cm was achieved. The importance of footplate in such immense bone defect and absent ankle joint can be appreciated in his demonstration. The extent and range of application of distraction osteogenesis has yet to be explored. This young patient showed high motivation and good compliance and we were able to successfully restore the tibial defect and further lengthening to correct limb discrepancy. Despite the duration of the treatment and the regular follow up the patient and his parents were satisfied with the results.

## Data Availability

The images and data sets used in the current study are available from the corresponding author on reasonable request.
